# ﻿Genus *Erica*: An identification aid version 4.00

**DOI:** 10.3897/phytokeys.241.117604

**Published:** 2024-04-24

**Authors:** E. G. H. Oliver, Nigel Forshaw, Inge M. Oliver, Fritz Volk, A. W. S. Schumann, Laurence J. Dorr, Rendert D. Hoekstra, Seth D. Musker, Nicolai M. Nürk, Michael D. Pirie, Anthony G. Rebelo

**Affiliations:** 1 Department of Botany and Zoology, University of Stellenbosch, Private Bag X1, 7602 Matieland, South Africa; 2 20 Oakridge Close, Oakridge, 7806, South Africa; 3 Stellenbosch, South Africa; 4 Greyton, South Africa; 5 Somerset West, South Africa; 6 National Museum of Natural History, Department of Botany, MRC-166, Smithsonian Institution, P.O. Box 37012, Washington, D.C, 20013-7012, USA; 7 Zeekoegat Homestead, Riversdale, 7760, South Africa; 8 Department of Biological Sciences, University of Cape Town, Private Bag, Rondebosch 7701, Cape Town, South Afric; 9 Department of Plant Systematics, Bayreuth Centre of Ecology and Environmental Research (BayCEER), University of Bayreuth, Universitätsstraße 30, 95447 Bayreuth, Germany; 10 University Museum, University of Bergen, Postboks 7800, NO-5020 Bergen, Norway; 11 Threatened Species Unit, South African National Biodiversity Institute, Cape Town, South Africa; 12 Pearson Chair of Botany, University of Cape Town, Cape Town, South Africa; † Deceased

**Keywords:** Ericaceae, GBIF, iNaturalist, species identification, World Flora Online

## Abstract

Species identification is fundamental to all aspects of biology and conservation. The process can be challenging, particularly in groups including many closely related or similar species. The problem is confounded by the absence of an up-to-date taxonomic revision, but even with such a resource all but professional botanists may struggle to recognise key species, presenting a substantial barrier to vital work such as surveys, threat assessments, and seed collection for ex situ conservation. Genus *Erica*: An Identification Aid is a tool to help both amateurs and professionals identify (using a limited number of accessible characteristics) and find information about the 851 species and many subspecific taxa of the genus *Erica*. We present an updated version 4.00, with new features including integrating distribution data from GBIF and iNaturalist, links to taxonomic resources through World Flora Online, and a probability function for identifications, that is freely available for PCs. It remains a work in progress: We discuss routes forward for collaboratively improving this resource.

## ﻿Introduction

Species are among the most basic units of biology and ecology, as fundamental as particles in physics and molecules in chemistry. The identification of individual organisms to a scientifically correct species name is of central importance, as it supports communication and allows for linking and extrapolating of information (Renner 2016). In large genera or recently radiated groups species identification is often challenging, because variation in phenotypes or diagnosable traits among species is often small. As a result, comprehensive taxonomic revisions that provide identification keys to all taxa are often not available for such groups, including large flowering plant genera (>500 species; Frodin 2004). Even when such information is available, it is often dispersed among multiple publications on individual subgroups. This is the case in the large flowering plant genus *Erica*, which has 851 currently recognised species (Elliott et al. 2024).

Whilst the relatively few European species of *Erica* are well documented (Nelson 2011), the many times more species found across Africa and Madagascar are much more challenging to identify. Traditional keys for South African species presented in Flora Capensis (Guthrie et al. 1905) and in the works of Dulfer (Dulfer 1964, 1965) are difficult to work with and do not include a substantial proportion of more recently described diversity. Some other works also provide keys and other identification tools for substantial numbers of species (Baker and Oliver 1967; Oliver 2000), or groups of similar or closely related species (Oliver and Oliver 2002, 2005), or those within particular regions (Oliver and Oliver 2000b; Beentje 2006). There are annotated checklists for South African species (Oliver and Oliver 2000a, 2003). Later editions of some of the historic literature, including stunning illustrations that accompanied some of the earliest original descriptions (Andrews 1802, 1845), are now available to view on Biodiversity Heritage Library (https://www.biodiversitylibrary.org/). A popular volume presents photos and short descriptions of many Cape species (Schumann et al. 1992) and another was recently published (Manning and Helme 2024). However, in general the literature is large, complex, and dispersed, and not all is openly or easily available. Given the large numbers of species, this presents a substantial challenge for anyone attempting to identify the numerous threatened *Erica* species (Raimondo et al. 2009), including conservationists who may not be specialists.

**Figure 1.**
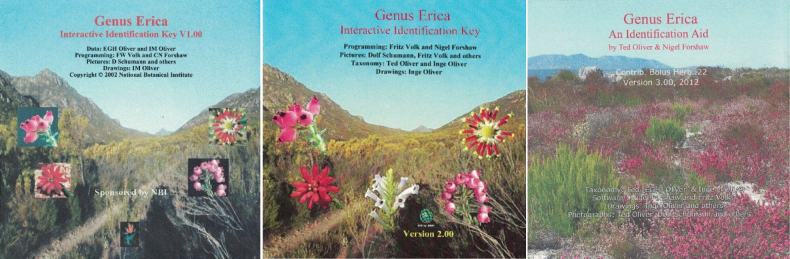
The publications of earlier versions of the *Erica* Identification Aid (formerly ‘Interactive Identification Key’). Note: version 2.00 was published as Contributions from the Bolus Herbarium vol. 22, and version 3.00 as volume 23 idem.

This was the motivation behind the development of the *Erica* Identification Aid (Oliver et al. 2002; Volk et al. 2005; Oliver and Forshaw 2012) (Fig. 1). FV, an amateur botanist, conceived the idea of a simple computer package to help identify South African Ericas, and collaborated with the preeminent taxonomic specialists EGHO and IMO who had formally described many species (Nelson et al. 2024) and were in the process of revising groups within the genus (Oliver, 2000; Oliver and Oliver 2002, 2005). The objective as stated in version 3.00 (Oliver and Forshaw 2012) was “to provide the *Erica* enthusiast with a simple aid to help identify Ericas, this based on as few simple characters as possible and all visible with a 10x magnifier, and in so doing, reducing the number of *Erica* species to a manageable number, among which the unknown *Erica* may be found. The detailed drawings and pictures may then be compared to the sample to enable the user to determine the species.”

**Figure 2.**
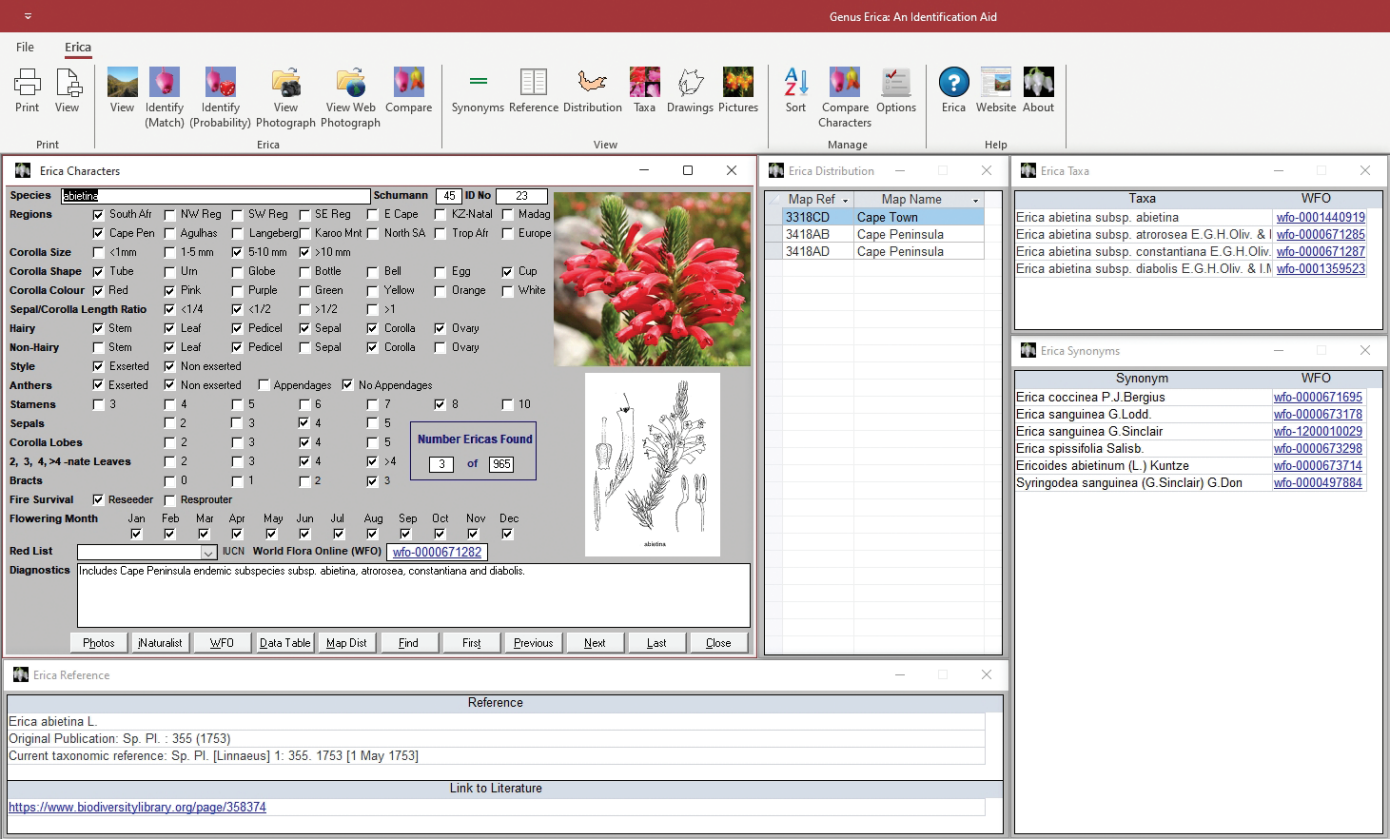
The main screen of the *Erica* ID aid in ‘view’ mode. The top ribbon provides access to, from left to right, the different identification and view modes; separate windows for information sources (open below the ribbon); options for presenting data in the aid; and links to the help file, webpage, and version information. The open windows, clockwise from top left are the characters used for narrowing down possible species identifications with options underneath for showing and finding data within the ID aid or through external links (‘map distributions’ opens Google Earth); distribution (a list of QDS map references in which the taxon is found); subspecific taxa; synonyms presented with World Flora Online (WFO) links; and references, including where available a DOI link to the relevant taxonomic treatment.

The characters in question are summarised in the main screen of the version 4.00 of the *Erica* ID aid (Fig. 2). In versions 1.00 and 2.00 these were corolla size, shape, and colour; hairiness of the stem, leaf, pedicel, sepal, corolla, and ovary; exertion or not of the style and of the anthers, the presence of anther appendages; numbers of stamens, sepals, corolla lobes, leaves in a whorl, and bracts. In version 3.00, sepal/corolla length ratio and whether there is evidence of resprouting from the base after fire were added. In addition, flowering month is coded, as is the geographic origin of specimens at global level as well as regionally within South Africa. The latter follows Goldblatt and Manning (2000) for regions within the Cape, whilst in South Africa outside the Cape distinction is drawn between ‘KZ-Natal’ (the province of KwaZulu-Natal) and ‘North South Africa’ (including Free State, Gauteng, Mpumalanga, Limpopo and North West provinces). Distribution and phenology are particularly informative given the high degree of regional endemism of taxa and the broad spread of flowering across the year in the Cape.

The authors developed the *Erica* ID aid using Microsoft Access as a deliberate alternative to dedicated software packages such as DELTA (Dallwitz 1992) which were available at the time but which they considered too complex to be easily accessible. FV did the proof of concept, initial data collection with encoding of data provided by EGHO and IMO and design of the system using the inbuilt functionality of Microsoft Access. NF joined the team in 2001 to develop the programming and added mapping of distributions, originally based on data from herbarium records at quarter degree squared (QDS) resolution (15' per side, approximating 25 km), projected using ESRI’s light weight data viewer ArcExplorer. FV took photos of herbarium material where those of live material were lacking. Many of the original photos were made available by AWSS from “Ericas of South Africa” (Schumann et al. 1992); others were added by EGHO, FV and others (see Acknowledgements). IMO provided numerous drawings and sketches with notes for South African Ericas. Many were intended for research and not to be of publishable quality. Further drawings were contributed by EGHO and several by other artists and extracted from the literature. The first version was released as a data CD (Oliver et al. 2002), second and third as volumes 22 and 23 of Contributions from the Bolus Herbarium (Volk et al. 2005; Oliver and Forshaw 2012; Fig. 1). At the publication of version 3.00, the *Erica* ID aid had incorporated around 15 years of development, containing data on 949 *Erica* species, subspecies and selected recent synonyms covering all the Southern African, Tropical African, Madagascan, Mascarene and European species recognised by the authors.

In the more than a decade since the release of version 3.00, the *Erica* ID aid had become increasingly incompatible with current software and in need of updating and further development. Our aim is to present a new version of the *Erica* ID aid that works on current PCs and continues to reflect the state of knowledge in *Erica* taxonomy. It should remain a useful tool for non-professionals, whilst incorporating more information from openly available sources such as World Flora Online (WFO; https://wfoplantlist.org/plant-list/) and the Global Biodiversity Information Facility (GBIF; https://www.gbif.org/) in a way that facilitates access to and improvement of the primary data, also for professional botanists.

## ﻿Features of the *Erica* identification aid and changes implemented in version 4.00

Versions 1.00–4.00 are all archived on Zenodo (https://zenodo.org/communities/erica?q=&f=subject%253Aspecies%20identification), including the full installation kit plus the raw data of each as plain text (.CSV). Version 4.00 is available here: https://doi.org/10.5281/zenodo.10407033, including alternative installation kits for those that have specific older versions of MS office installed (any should work in the absence of an MS office installation).

Unless specified, features of version 3.00 are maintained in version 4.00. These include View (all data); Identify (strict matching); Compare (selected characters, diagnostics, photos, or illustrations for two or more taxa); Sort (by taxon, by Schumann and/or by ID number, the latter two reflecting subgeneric classifications); Map (of QDS) was projected using ArcExplorer and is now presented with Google Earth (see below), whilst it is also possible to generate lists of taxa per QDS. A detailed help file is maintained with minor updates. It includes helpful descriptions of characters and a detailed description of the origins of the aid and people involved.

### ﻿Changes implemented in version 4.00

Identification:

• A new probability algorithm as an optional alternative to strict matching of characters, with easy switching between the two, to find taxa with consistent characters or minor mismatches.

The probability algorithm works as follows: Given 21 probability “Groups”, corresponding to the characters with different coded states (including geographic distribution – “regions” – and flowering month, as well as morphological attributes, as listed above), the probability of a given identification is the sum of the probabilities for all the Groups that the user employs. The probability for each component in the Group is calculated to be 1 divided by the number of species that have the specific character state. For example, observing a tubular flowered *Erica* on the Cape Peninsula: there are 359 *Ericas* which are coded as corolla shape “tube”, so the “Corolla Shape” Group contributes 1/361 = 0.0027700 to the sum of all Group probabilities, and there are 116 Ericas which are coded as present on the Cape Peninsula, so the “Regions” Group contributes 1/115 = 0.0086956. In this case, the sum of probabilities of each Group is 0.0027700 + 0.0086956 for every *Erica* coded as Tube and Cape Peninsula. The sum of all probabilities is calculated for all Ericas, and these are displayed in descending order of probability, with those of equal overall probability listed alphabetically. The match algorithm treats coding as “OR” within a Group, as opposed to “AND”, thus reflecting the variation that is common within species but unlikely to be represented in a single specimen. If more than one state is selected by the user, this is treated as uncertainty. The probability for that group is divided by a greater number of taxa exhibiting one or other state and therefore correspondingly reduced, as opposed to dividing by the smaller number of taxa that exhibit both. By considering the prevalence of individual character states, those that are rare will impart a greater probability than those that are more common. The probability algorithm will also be less sensitive to missing data - characters that have yet to be coded for particular taxa (see below) - than strict matching.

Taxonomy and nomenclature:

- As of the WFO public release on 22 ^nd^ of December 2023, the *Erica* ID is synchronised with WFO, representing all 851 species of *Erica* currently accepted on WFO (the 852 in the December 2023 public snapshot wrongly includes *E.perlata* G.Sinclair as Accepted instead of as Unplaced following Nelson et al. 2023), presenting for each accepted species a comprehensive synonymy (as opposed to only recently used names) plus references and links to taxa and taxonomic literature.
- Representation of inclusive species, e.g. *Ericaabietina* or *E.banksii* (as well as the subspecific taxa of such species that were originally presented).
- Representation of additional subspecific taxa not covered in v. 3.00 but included in SANBI’s list for threat status assessment and treated as accepted on WFO, by listing and mapping (see below).
- Other *Erica* names: WFO includes numerous published names categorised as ‘Unplaced’ which cannot be attributed to known taxa but may be encountered in the literature. Such names are not presented openly in the ID aid but can be found when searching for names under ‘find’, with an explanation and WFO link for further information.
- Threat status (South African species based on Raimondo et al. (2009), represented by IUCN categories).
- Additional data collection for species, including both character coding and new images. This remains incomplete: a summary by character and region is presented in Appendix 1.

**Figure 3.**
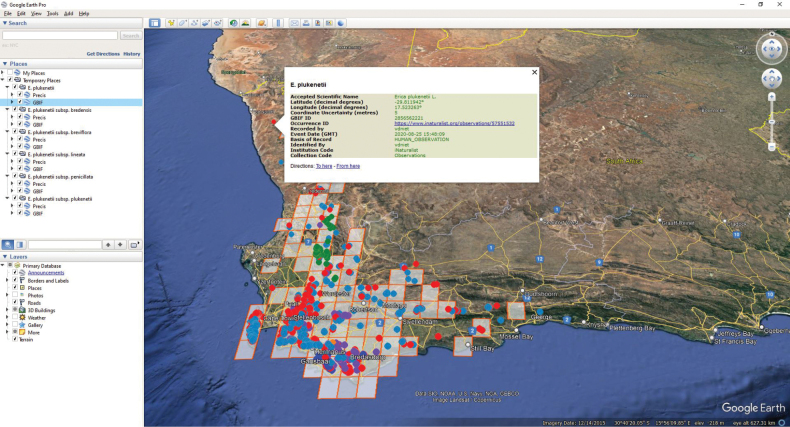
Distribution data presented on a Google Earth map. The Quarter Degree Square (QDS) map references listed in the main aid are projected as grid squares with separate individual dots for openly available GBIF data in different colours for different taxa displayed. In this example, on clicking ‘map distributions’ whilst viewing the inclusive species *Ericaplukenetii*, data points representing observations/specimens determined to species only are presented in red, with those determined to the five different subspecies presented in orange (ssp. bredensis), green (ssp. breviflora), dark blue (ssp. lineata), purple (ssp. penicillata), and light blue (ssp. plukenetii) respectively. On clicking on an individual dot, the underlying information is shown, including links where available, such as in this case an iNaturalist observation. Note that the QDS grid data derived from version 3.00 does not cover all the point data, and that there also isn’t point data representing all the QDS. This could indicate errors and/or gaps in the data that are worth further investigation.

Distributions:

- Augmenting the original PRECIS-based distribution summaries at quarter degree square (QDS) resolution with overlaid point localities curated from GBIF data (GBIF.org 2023).
- Presentation on Google Earth, with accessible metadata including links to original sources, where available (Fig. 3). A default of 5,000 records are projected (this can be changed under ‘options’). This represents all the curated data for most taxa, whilst restricting the data to that which can be processed with modest random-access memory (RAM) for the most documented (European) species.
- Mapping of inclusive species (i.e. specimens/observations not identified to subspecies) as well as of subspecific taxa.
- Links to observations by taxa on iNaturalist (https://www.inaturalist.org).

The *Erica* ID aid runs on any Windows 10 or Windows 11 PC and uses 502 MB of disk space.

## ﻿Discussion

Since the original versions of the *Erica* ID aid, development of platforms such as iNaturalist, the Biodiversity Heritage Library, and GBIF have dramatically increased open access to information on biological diversity, including photos, literature, and even machine learning based species identification. The latter is currently only trained on around 200 species (represented by >100 photos), and it does not resolve subspecific ranks. Online access can reduce the need for resources within any dedicated identification tool such as the *Erica* ID aid itself. On the other hand, it is increasingly challenging to filter and quality control the sheer volumes of accessible data online.

As part of the ongoing development of the *Erica* ID aid, we set out to improve both accessibility and quality of existing online resources for *Erica*. We shifted from maintaining a limited names database within the *Erica* ID aid to improving at source and synchronising with openly available data for *Erica* names and literature through contributing to the development of the World Flora Online (WFO 2023, Elliott et al. 2024). Links to WFO within the ID aid lead to further links to many taxon-specific online resources.

We incorporated links to iNaturalist data both for species and via GBIF to individual observations. The latter is through a curated dataset downloaded from GBIF representing data from a wider range of sources, including herbarium records, and has been filtered to reduce noise as a resource particularly for conservation prioritisation (Pirie et al. 2024). That filtering process can be repeated in the future with a fresh download of updated GBIF data, which will be further expediated when WFO identifiers are associated with those on GBiF (e.g., through integration of WFO with the Catalogue of Life checklist; Elliott et al. 2024). In both cases, it is possible for anyone not just to see countless recent photos of species, but also to spot and potentially correct at source remaining errors – and even gaps – in this primary data, which may then be incorporated in the next *Erica* ID aid data refresh. By linking to primary data (WFO; GBIF), improvements and additions can be incorporated in automated fashion on a regular basis. We anticipate mirroring the WFO 6-monthly public snapshots.

Many of the photos provided in earlier versions of the ID aid were compromised in resolution due to both the limitations of storage media and quality of images available. Those provided from ‘Ericas of South Africa’ (Schumann et al. 1992) are often visibly pixelated, having been scanned from the book rather than from the original slides. They were already in the process of being replaced. The originals have not been digitised and are archived within the substantial wider collection of AWSS slides in the Compton Herbarium at Kirstenbosch. Photos within the *Erica* ID aid are arguably less important now that so much is available online, but we are nevertheless maintaining and supplementing confirmed and informative images as a useful reference within the overview of characters, distributions, and names. Selected images will be added through a collective effort using iNaturalist, e.g. enlisting contributors through the project ‘Ericas of Southern Africa’ (https://www.inaturalist.org/projects/ericas-of-southern-africa). The sketches of IMO, presented next to the photos, remain an unparalleled resource.

### ﻿Limitations and future development

In version 3.00 a limitation of the *Erica* ID aid was clearly stated which remains in this new version: “Due to the limited number of characters in this package, use of the Diagnostics & notes may have to be resorted to in order to come to a final decision, but this aspect is far from complete due to the vast number of species that still need to be dealt with” (Oliver and Forshaw 2012). We do not anticipate coding more characters across the genus, and so diagnoses that refer to other characteristics and discern the most similar species will continue to be important in many cases.

Even the current limited numbers of characters are yet to be completely coded for all the taxa in the ID aid. Some characters and regions are better known than others. Data for flowering period, and for Tropical African and particularly Madagascan taxa are very incomplete (Appendix 1). It may be possible to automate the updating/checking of phenology data by analysing GBIF/iNaturalist data. Species of *Erica* can be identified for a considerable period after flowering because the flowers persist on the plants. Nevertheless, observations may tend to be of specimens at or around flowering, and outliers could be identified and potentially flagged, adding to the primary metadata for individual observations, whilst assessing and rectifying any hard inconsistencies with current coding within the aid. Flowering state can be annotated directly on iNaturalist observations using a curation tool, potentially by volunteers. We should prioritise work on threatened species to support effective conservation action.

IUCN threat status is derived from SANBI’s seminal work (Raimondo et al. 2009), for which Ross Turner assessed numerous species of *Erica*. It is now in urgent need of updating. Observations of threatened species, e.g., through the iNaturalist platform, are particularly valuable for informing conservation efforts. The South African RedList project on iNaturalist (https://www.inaturalist.org/projects/redlist-s-afr) also facilitates documenting population sizes and threats by citizen scientists. These will need to be supplemented by formal assessments of localities as a whole, as is ongoing under the Custodians of Rare and Endangered Wildflowers (CREW) programme (https://www.sanbi.org/biodiversity/building-knowledge/biodiversity-monitoring-assessment/custodians-of-rare-and-endangered-wildflowers-crew-programme/).

The current implementation, on Windows PCs only, is obviously not ideal: users might wish to use this tool on different platforms, including on mobile devices. This would be an important future step in development of the *Erica* ID aid, that will be made easier by our archiving and use of openly accessible data.

## ﻿Conclusions

Tools for species identification are essential for effective conservation efforts, particularly in species rich genera such as *Erica*. With this new version, the invaluable *Erica* Identification Aid is updated and openly available, with new features that improve its functionality and integrate online resources such as WFO, GBIF, and iNaturalist and offer scope for wider contributions to improving that primary data.

In the absence of Ted and Inge Oliver as a single hub for *Erica* taxonomy, it is our hope that future efforts can be spread among a broad group of collaborators, such as coordinated through the Global Conservation Consortium for *Erica* (Pirie et al. 2022). NF maintains a tracking tool that allows different people to edit the *Erica* ID aid in parallel and to audit the resulting updates. This can facilitate contributions from experts in local floras whilst minimising the burden of coordination. The ID aid is still very much in development, and targeted improvements will be needed, such as through prioritising the effective identification of threatened species for conservation.

Humphreys et al. (2019) wrote: “We urge botanists to compile data on search effort, species density, abundance and detectability and to engage local people in the search for their missing biodiversity. Such efforts will improve our understanding of genuine extinctions and help target future conservation action”. We believe that tools such as the ID aid presented here will help towards this aim.

## ﻿Additional information

### Author ORCIDs

Nigel Forshaw https://orcid.org/0009-0000-4782-1875

Laurence J. Dorr https://orcid.org/0000-0001-7157-363X

Rendert D. Hoekstra https://orcid.org/0009-0001-1506-7107

Seth D. Musker https://orcid.org/0000-0002-1456-1373

Nicolai M. Nürk https://orcid.org/0000-0002-0471-644X

Michael D. Pirie https://orcid.org/0000-0000-0000-0000

Anthony G. Rebelo https://orcid.org/0000-0003-0403-4470

### Data availability

All of the data that support the findings of this study are available in the main text.

## ﻿Appendix 1

**Table A1.** Overview of completeness of character coding by region. Note that the total number of Ericas includes all 851 recognised species plus many (but not all) subspecific taxa. Threat status has been assessed in South Africa at the lowest taxonomic level only, so the numbers for non-assessed taxa include many inclusive species for which subspecific taxa have been assessed individually.

Criteria	Total	South Africa	Agulhas Plain Region	Cape Peninsula	Eastern Cape	Karoo Moun-tain Region	KWA-Zulu Natal	Langeberg Region	North West Region	North South Africa	South East Region	South West Region	Tropical Africa	Mada-gascar	Europe
Total number of Ericas	961	848	103	115	26	119	37	140	167	17	120	467	41	45	28
Missing corolla size	63	15	2	0	2	1	1	1	0	0	3	5	7	35	6
Missing corolla shape	69	14	1	0	1	2	1	1	0	0	3	4	14	35	6
Missing sepal/corolla length ratio	53	15	0	0	2	0	2	1	1	1	0	6	18	11	9
Missing stem hairiness	68	20	2	0	2	1	1	1	0	0	2	9	5	36	7
Missing leaf hairiness	66	20	2	0	2	1	1	1	1	0	1	10	7	35	4
Missing pedicel hairiness	70	20	2	0	2	1	1	1	0	0	2	9	9	33	8
Missing sepal hairiness	74	21	2	0	2	1	1	1	0	0	2	10	11	34	8
Missing corolla hairiness	66	16	2	0	2	1	1	1	0	0	1	7	11	35	4
Missing ovary hairiness	70	21	4	2	2	0	1	1	2	0	3	10	7	36	6
Missing style exsertion/inclusion	76	20	1	0	2	1	1	1	0	0	4	7	11	34	11
Missing anthers exsertion/inclusion	72	17	1	0	1	1	1	1	0	0	3	7	14	36	5
Missing appendage presence/absence	73	20	1	0	2	2	1	1	0	0	3	8	14	34	5
Missing number of stamens	63	15	1	0	2	0	1	0	1	0	1	7	10	34	4
Missing number of sepals	63	15	1	0	2	1	1	0	1	0	2	6	7	37	4
Missing number of corolla lobes	62	16	2	0	2	1	1	0	1	0	2	7	7	36	3
Missing -nate leaves	67	22	2	1	2	1	1	0	0	0	4	11	6	35	4
Missing number of bracts	62	15	1	0	2	1	1	0	1	0	2	6	10	33	4
Missing fire survival strategy	6	6	0	0	3	0	1	0	0	1	0	1	0	0	0
Missing flowering month	102	29	2	0	2	2	2	0	1	1	4	14	22	42	9
Missing IUCN Red List category	242	145	15	25	14	19	25	23	30	9	26	69	31	45	21
